# Epidemiology of injuries at the first transplant football world cup

**DOI:** 10.3389/fspor.2025.1685440

**Published:** 2025-11-19

**Authors:** Valentina Totti, Filippo Ferrari, Simone Paltrinieri, Giovanni Vitale, Giovanni Mosconi, Alessandro Nanni Costa, Gianluigi Sella, Maria Cristina Morelli, Paolo Caraceni, Giulio Sergio Roi

**Affiliations:** 1 Internal Medicine Unit for the Treatment of Severe Organ Failure, IRCCS Azienda Ospedaliero-Universitaria di Bologna, Bologna, Italy; 2 Post-graduated School of Sports Medicine and Physical Exercise, University of Bologna, Bologna, Italy; 3 Fondazione Policlinico Sant’Orsola, IRCCS Azienda Ospedaliero-Universitaria di Bologna, Bologna, Italy; 4 IRCCS Azienda Ospedaliero-Universitaria di Bologna, Bologna, Italy

**Keywords:** incidence, kidney, sport, injury patterns, football, transplant recipients

## Abstract

**Introduction:**

Recreational football is widely recognized as a health-promoting activity, with proven long-term benefits for physical and mental well-being. For transplant recipients, it offers additional value by supporting recovery, reintegration, and quality of life. To promote safe participation in sport among this population, the World Transplant Games Federation launched the 1st World Transplant Football Cup. This study aimed to investigate the epidemiology of injuries sustained during the tournament.

**Methods:**

Matches were conducted according to modified FIFA 7-a-side rules, tailored to ensure the safety of transplanted organs and tissues. Each team included up to 16 players of any age or gender. Matches lasted 20 min, with unlimited substitutions. Games were played on two standard 60 × 40 m pitches with 3 m goals. Medical coverage was ensured by two sports physicians present throughout the event. Injury data were collected using a standardized form for all requests for medical intervention (RfMI).

**Results:**

A total of 172 transplant recipients participated (kidney: *n* = 103; liver: *n* = 25; bone marrow: *n* = 18; heart: *n* = 14; kidney-pancreas: *n* = 7; lung: *n* = 5), representing 11 teams. Across 35 matches (total exposure: 326 h and 40 min), 28 RfMIs were reported (16% of participants). Players (mean age 36.0 ± 11.1 years) included 26 males and 2 females, distributed across all field positions. Injury mechanisms were non-contact (43%), indirect contact (32%), and direct contact (25%). Most injuries were acute (82%), followed by acute-on-chronic (12%) and chronic (4%). The most frequent injury types were contusions (32%), sprains (21%), muscle strains (18%), and abrasions (14%), with less common events including tendinopathies, minor concussions, and toe infections (4%). The incidence of RfMI was 85.7 per 1,000 h (95% CI: 54.0–117.4), and time-loss injuries (*n* = 17) occurred at a rate of 52.0 per 1,000 h (95% CI: 27.3–76.8). All injuries were managed on site, except one which required hospital care.

**Conclusions:**

Despite a moderate incidence of injury, the majority were minor and no transplant-related injuries were recorded. These findings support the safety and feasibility of recreational football in transplant recipients, reinforcing its role as a beneficial component of long-term health promotion.

## Introduction

Football (soccer) is one of the most widely played sports worldwide, with over 500 million participants. Engaging in recreational football regularly—about three times per week—is considered one of the best long-term strategies for maintaining good health, as it can help lower blood pressure, reduce body fat and blood levels, increase muscle mass, and improve cardiovascular risk profiles (1–3). Additionally, football is a highly motivating and enjoyable leisure activity that attracts a large portion of the population, making it a valuable health-promoting option even for transplant recipients.

Transplant recipients who play football have shown energy expenditures and levels of activity comparable with healthy football players; they were able to perform physical activity at an intensity level greater than three multiples of the basal metabolic rate (BMR), as well as healthy controls practicing football (4). Moreover, organ-transplant patients have shown significant quality-of-life and functional gains when engaging in structured physical activity and sports (4).

On the other hand, it is well known that after transplantation, patients show a chronic reduction in peak oxygen uptake, associated with the decrease in muscle mass and muscle quality due to immunosuppressive therapy interactions (5).

Promotion of sports activity after transplantation is not a common practice. The lack of published evidence-based practice for transplant recipients within “sports therapy” also appears to exist for sport training advice. In a study conducted on transplanted athletes participating in national and international transplant games, only 57% received specific training guidelines to undertake sports (5). Furthermore, over half of the participants perceived limitations preventing them from performing at their true potential. Of these, the main factors were current injury or illness (23%), lack of fitness/strength (18%), fear of overdoing it and lack of motivation (13%) (5). Instead, another study, evaluating transplanted athletes attending the World Transplant Games 2023, showed that these athletes reported fewer barriers related to physical limitations, fear of exercise, and comorbidities than non-sports transplant recipients (6). Transplanted athletes also reported a wider range of facilitators, often associated with the pleasure and positive psychological effects they derive from participating and competing in their chosen sport with high levels of support from their social networks and family (6).

However, transplanted subjects are more fragile than the healthy general population, and this must be considered. Sport in organ transplant recipients, when practiced at strenuous intensities, may have potential adverse events from musculoskeletal (e.g., stress fractures, tendinopathies), immunological (e.g., increased risk of infection), cardiovascular (e.g., cardiovascular events during acute strenuous exercise), endocrine (e.g., dysregulation of blood glucose levels) and renal (e.g., impaired kidney injury) perspective (7). However, these medical conditions can also affect healthy athletes, highlighting the risks of sport participation for anyone (8).

Nevertheless, scientific literature confirms the beneficial effects of physical and sporting activities not only for sedentary people, but also on the transplanted organ function, in particular on glucose and lipid metabolism and aerobic capacity, as well as on quality of life (9–11). Exercise and sports therapy are considered a non-pharmacological treatment for the prevention of cardiovascular and metabolic events that represent the leading cause of death after transplantation (12–14). Chronic immunosuppressants and glucocorticoids used by transplant recipients increase the potential for osteoporosis and fractures, whether involved in sport or not (6, 15). Lastly, sport has a positive impact on complete social reintegration and improves the perception of quality of life (11). From all this, it follows that discouraging the promotion of sporting activities in transplanted patients seems to be anachronistic, provided that there is a good organ or tissue compatibility and a good tolerance to the immunosuppressive therapy (16).

In terms of traumatic risk, football is commonly regarded as a contact sport, although in some respects it approaches collision sports. Knowledge of epidemiological data makes it possible to apply suitable preventive principles to curb the growth of traumatic events.

Studies on football traumatology show that the most common type of injury grouping was muscle/tendon, followed by contusions in the lower extremities, and very rarely in the locations where a transplanted organ is present (abdominal or chest area) (17–19).

Hence, we explored the requests for medical intervention during the first Transplant Football World Cup with the aim to contribute to the discussion about the risks of injuries affecting transplanted patients during a contact sport such as football.

## Materials and methods

The first Transplant Football World Cup (TFWC) took place in Cervia, Italy, from 8th to 14th September 2024. The competition format consisted of two groups – Group A with six teams and Group B with five – determined during a live draw earlier in the week. The top two teams from each group advanced to the knockout rounds, while the remaining teams competed for placement positions 5th through 11th. All eleven competing teams took to the field in five days. The TFWC was promoted by the World Transplant Games Federation, organized by AiCS – Associazione Italiana Cultura Sport, and made possible thanks to the Ministry for Sport and Youth through the Department for Sport.

Modelled on the FIFA World Cup, this tournament brought transplant and bone marrow recipients from eleven countries (Australia, Chile, England, France, Ireland, Italy, Northern Ireland, Romania, Spain, USA and Wales), to compete in a 7-a-side format. All matches were refereed by AiCS officials, with special rules designed to protect the health of the players.

Gatekeeper consent was provided from the World Transplant Games Federation, the AiCS officials and national team managers as part of the institutional ethics process, in accordance with 1964 Helsinki declaration involving human participants and the STROBE (Strengthening the Reporting of Observational studies in Epidemiology) guidelines (20). All transplant football players met the required conditions for competition, that is, having received one or more life-supporting allograft (kidney, liver, heart, bone marrow, lung, pancreas, or combined), being more than six months post-transplant, and with a stable allograft and medically fit, as signed off by their physician.

Two standard FIFA pitches measuring 60 × 40 m, with 3 m goals, were utilized. Teams consisted of a maximum of 16 players of all ages and genders. Matches lasted 20 min with unlimited substitutions. Two sports physicians were always present to provide medical assistance and real-time monitoring of all requests for medical intervention (RfMI). Following the Injury Consensus Group of FIFA's Centre for Medical Evaluation and Research (21) detailed data were collected, together with the type of transplant, on:

- injury type,
- injury severity
- time-loss injury,
- injury mechanism.

The injury report form was used to collect information on any request for RfMI. Injuries were classified by the Orchard Sports Injury Classification System (OSICS).

Injury incidence was calculated as the number of injuries per 1,000 player-hours, with 95% confidence intervals (Cis).

**Figure 1:**
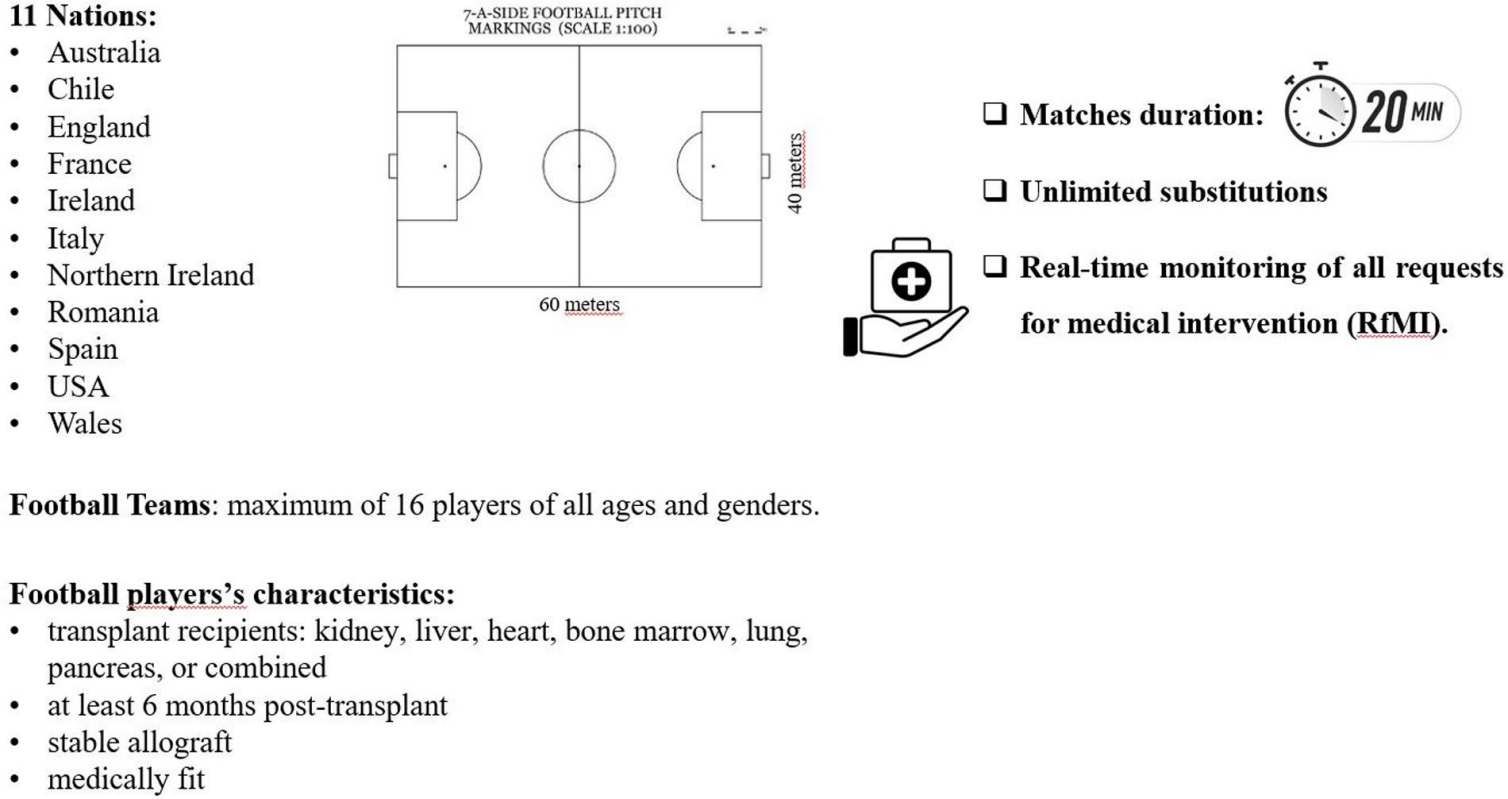
Competition rules and the study design.

The competition rules and the study design were shown in Figure 1.

## Results

Eleven teams participated, involving 172 transplanted kidney players (103; 60%), liver (25; 15%), bone marrow (18; 10%), heart (14; 8%), kidney-pancreas (7; 4%), and lung (5; 3%) with mean age of 41.7 ± 10.8 years; 170 were male and 2 were female (Table 1). Thirty-five matches were played, corresponding to 326 h and 40 min of exposure.

**Table 1:** Characteristics of football players and the RfMI according to the type of transplant among participants in the 1st TFWC.

Type of transplant	Players	RfMI
Kidney	103 (60%)	9 (9%)
Liver	25 (15%)	13 (52%)
Bone marrow	18 (10%)	2 (11%)
Heart	14 (8%)	3 (21%)
Kidney-pancreas	7 (4%)	1 (4%)
Lung	5 (3%)	0 (3%)
***ALL***	***172 (100%)***	***28 (100%)***
Age (years), mean(±SD)	41.7 ± 10.8	
Sex	170 male	2 female

**Figure 2:**
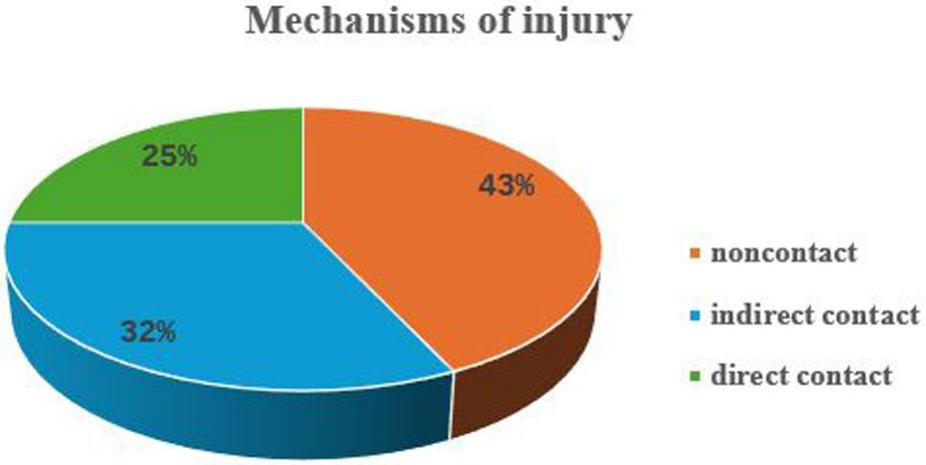
Mechanisms of injuries (%) occurred during the 1st TFWC.

RfMIs were 28 (16% of participants) involving 13 liver (52%), 9 kidney (9%), 3 heart (21%), 2 bone-marrow (11%) and one kidney-pancreas (14%) recipients. Among them, 26 were males and two females. They were aged 36.0 ± 11.1 years, playing as defenders (10), strikers (8), midfielders (6), and goalkeepers (4). Mechanisms of injury were by noncontact (12), indirect contact (9) and direct contact (7) (Figure 2). Injuries were classified as: acute (23; 82%), acute- on-chronic (4; 12%) and chronic (1; 4%). Injury types were shown in Table 2. The types of injuries in relation to the role played on the field are shown in Table 3, and the locations of injuries were shown in Table 4.

**Table 2:** Classification of injuries that occurred during the 1st TFWC.

Injuries classification	N°	%
Acute	23	82%
Acute on chronic	4	12%
Chronic	1	4%
Injury type		
Contusions	9	32%
Sprains	6	21%
Muscle strain	5	18%
Abrasions	4	14%
Foot tendinopathy	1	4%
Wound from shoe studs	1	4%
Minor concussion	1	4%
Big toe infection	1	4%

**Table 3:** Types of injuries in relation to the roles played during the 1st TFWC.

Injury type	Roles			
	Defenders	Strikers	Midfielders	Goalkeepers
Contusions	4	3	1	1
Sprains	3	-	3	–
Muscle strain	2	1	1	1
Abrasions	–	3	–	1
Foot tendinopathy	1	–	–	–
Minor concussion	–	–	1	–
Big toe infection	–	–	–	1
Wound from shoe studs	–	1	–	–
**Total injuries by role**	**10**	**8**	**6**	**4**

**Table 4:** The location of injuries during the 1st TFWC.

Injury location	Body part	*N*	Injury type
Head neck (4%)	Head	1	Concussion
Upper limb (7%)	Elbow	1	Abrasion
	Forearm	1	Contusion
Trunk (11%)	Abdomen	2	Contusion
	Chest	1	Contusion
Lower limb (79%)	Ankle	7	Sprain (6), contusion (1)
	Hamstrings	5	Muscle strain
	Knee	3	Abrasion
	Foot	2	Tendinopathy (1), infection (1)
	Hip	2	Contusion
	Thigh	2	Contusion
	Leg	1	Contusion
	**Total**	**28**	

Seventy-nine per cent of the injuries affected the lower extremities. Muscle strain, ligament sprain and contusion were the most common injury types. The knee, ankle and hamstring muscles were the most common injury locations. The RfMI incidence was 85.7 per 1,000 h (95% CI: 54.0–117.4); time-loss injuries (TLI) were 17, with an incidence of 52.0 per 1,000 h (95% CI: 27.3–76.8). All injuries were treated on site except for one wound requiring hospital referral. Despite the high incidence of TLI, only four injuries were of moderate severity (8–28 days off) and no injuries to the transplanted organs were reported.

## Discussion

To our knowledge, this is the first study to provide valuable insights into the incidence and characteristics of injuries sustained by transplanted organ recipients participating in a competitive sports football championship. In our study, the overall injury incidence rate was higher than that reported for professional players, i.e., 85.7 vs. 36.0 injuries per 1,000 h of exposure and 52.0 vs. 8.1 per 1,000 h of time-loss injuries (22). Given the uniqueness of the TFWC event and the type of athletes, this is probably due to the fact that we registered all the RfMI, regardless of whether it was an injury according to the definition of sports injury (23).

Most injuries were treated on site and were of mild to moderate severity, with only four injuries causing absence from play for 8 to 28 days. Significantly, no injuries directly affected the transplanted organs, highlighting that participation in competitive sports, under appropriate medical and coach supervision, may be safe for transplanted athletes.

Notably, liver recipients accounted for over half of the injuries, despite representing a smaller proportion of the total population. However, it is important to note that most of the RfMI came from players who played as defenders, and among these, most had undergone liver transplants (10 out of 13).

Most injuries were acute (82%) and affected the lower limbs, as reported in the UEFA injury study. Contusions, sprains, and muscle strains were the most common injury types (22). This distribution is typical in sports involving sudden changes of direction and physical contact. Chronic and acute-on-chronic injuries were relatively uncommon, indicating that many participants effectively managed their physical fitness well throughout the competition.

Injury patterns varied somewhat by playing position. Defenders and strikers suffered mainly contusions and sprains, while midfielders had a mix of sprains and minor concussions. Goalkeepers experienced a wider variety of injuries, including infection and abrasions, possibly related to their unique role and movements on the field. These findings underscore the importance of position-specific preventive strategies and conditioning programs tailored to the physical demands and injury risks of each role, which need further investigation.

Predisposing risk factors for injuries include age, body mass index, health, physical fitness, training level, excessive loading, insufficient recovery and underpreparedness. Although less than one-third of transplanted athletes considered they could train at a similar intensity as non-transplant recipient athletes, over half felt they recovered as well (5).

In the transplant community, many local football teams include transplant recipients as active players. Members of the national transplant football teams competing in the TFWC usually play alongside non-transplant teammates in various local leagues. Similar to national teams worldwide, these transplant athletes regularly come together for training sessions and participate in different championships.

Nonetheless, in some countries (e.g., Italy) the mandatory pre-participation screening for sport does not allow transplant recipients to compete in the so-called contact sports, football included. In particular, for kidney recipients it is supposed a hypothetical risk of injury to the abdominal area, where the transplanted kidney is located, while literature on other transplanted organs related to football is lacking.

In the present study, most injuries have been minor, supporting the idea that physical activity, even in competitive settings, can be safe and beneficial for well-managed transplant recipients. For kidney transplant athletes, regarding the incidence of kidney injuries in football is rarely reported as an isolated event, and kidney injuries are highly uncommon compared to other types of sports-related injuries, often grouped under broader categories such as abdominal or genitourinary trauma.

In a large study conducted by Grinsell et al. (24), which examined over 4.4 million athlete exposures, 23,666 injuries were recorded. Among these, only 18 kidney injuries occurred (i.e., 0.08%), none of which were catastrophic or required surgery. This contrasts with 3,450 knee injuries, 2,069 head/neck/spine injuries, 1,219 mild traumatic brain injuries, 148 eye injuries, and 17 testicular injuries. The majority of kidney injuries occurred in American football players (12 injuries). Sport-specific rates of kidney injuries were significantly lower than those of mild traumatic brain injuries, head/neck/spine injuries, and knee injuries across all sports.

From these data, it appear that kidney injuries occur far less frequently than other sports injuries, and this evidence does not support restricting sports participation for athletes with a single kidney (25) or those who have received a transplant (24). It is important to note that the transplanted kidney is placed in the iliac fossa rather than its natural retroperitoneal location, which may make it more vulnerable to trauma. However, current literature on this topic is limited. In our study, no injuries to transplanted kidney area were reported. Nonetheless, further long-term studies involving transplant recipients engaged in sports are necessary to better understand potential trauma-related risks. Football generally involves less direct abdominal contact compared to sports like American football or rugby. Available research indicates that abdominal injuries account for less than 5% of all football injuries (26). A study of professional players estimated that abdominal injuries make up approximately 2%–4% of all sports injuries with an incidence of 0.4 per 1,000 h of playing time at professional level, always without catastrophic consequences (22).

This study has several limitations. The sample size was relatively small, which may limit the generalizability of the findings. Furthermore, the rules of play were modified specifically to reduce injury risk. Lastly, the high incidence of RfMIs was due to the frequent requests for medical intervention during the matches, which may have led to an overestimation of the overall injury incidence.

Future studies could focus on longitudinal follow-up and on the role of conditioning, protective equipment, and sport training guidelines to further enhance safety and performance in this unique yet heterogeneous population, composed of individuals with different transplanted organs or tissues.

In conclusion, these findings contribute to the growing body of evidence supporting the physical and psychosocial benefits of sports participation in transplant recipients. However, the relatively high rate of RfMI suggests the need for ongoing monitoring and tailored preventive measures, although the risks for a recreational footballer represent far less danger than the much greater threat posed by a lack of exercise.

## Data Availability

The original contributions presented in the study are included in the article/Supplementary Material, further inquiries can be directed to the corresponding author.
